# Supplementary material for: Real World Performance of an Individualized Antitachycardia Pacing Algorithm
Source: J Cardiovasc Electrophysiol. 2025 Jun 4;36(8):1875–85. doi: 10.1111/jce.16747 (PMC12337625; doi:10.1111/jce.16747)
Supplement: Supplementary file 1 — Real World Indivdualized ATP Performance Supplemental Material JCE Rev02. [file JCE-36-1875-s001.docx]

**Supplemental Material: Real World Performance of an Individualized Antitachycardia Pacing Algorithm (IATP)**

*Assignment of Indication for Device*

For devices with available demographic data, an indication for the device was assigned based on a hierarchical algorithm. Key terms identifying secondary prevention criteria were given highest priority, followed by specific primary prevention terms, then inferences from more general terms. The specific terms are provided in Table S1.

*VT and VF Zone Detection Programming*

The span of VT detection intervals was broad, ranging from 300ms to 600ms and is detailed in Figure S1. Changes to a longer VT zone cutoff were observed in 35 patients (13%). The number of intervals to detect VT also spanned the minimum and maximum programmable values in the device. The guideline directed value of 24 was used in 18% of programming configurations. The contribution of MVT episodes for each VT NID value is provided in Figure S2. Changes to a larger NID were seen in 14 patients.

The detection programming of the VF zone was less varied than the VT zone. The longest VF zone cutoff was 360 ms and the shortest was 250 ms. The most common VF zone cutoffs were 300ms and 320ms. The full distribution of VF zone programming is provided in Figure S3. There were two patients with a change to a longer VF zone cutoff. For the number of intervals to detect, the nominal value of 30/40 intervals dominated, with 80% of configurations set to that value. There were no changes to VF NID. Despite guideline recommendations there were some shorter NIDs of 18/24 and 12/16. The number of episodes detected at each value of VF NID is shown in Figure S4.

*FVT Sub-zone Programming*

There were 46 patients that had 139 episodes of MVT occur in a Fast VT sub-zone, the majority of which (103 episodes from 34 patients) were in the FVT via VF configuration. The FVT via VT configuration was only used in seven patients, with 19 episodes of MVT. The FVT zone cutoffs used are provided in Table S2.

*Therapy Programming*

For the VF zone, only a single ATP therapy can be selected, so when IATP was applied in the VF zone it was as the first and only ATP therapy in the zone. For the FVT and VT zones, IATP could also be used as a later therapy. In the FVT zone, one patient also had IATP as the second therapy which was applied in one episode. In the VT zone, first and second therapy set as IATP was found in 19 episodes from 10 patients. A single patient had IATP set to the first three therapies in the VT zone in 30 episodes.

The programming of the Minimum S2/S3 parameter was dominated by nominal values in each zone. The details of zone-based programming are in Table S3.

**FIGURES**

**Figure S1:**

Figure S1: Histogram of the programmed values of the VT Detection Interval in the devices with MVT episodes.

**Figure S2:**


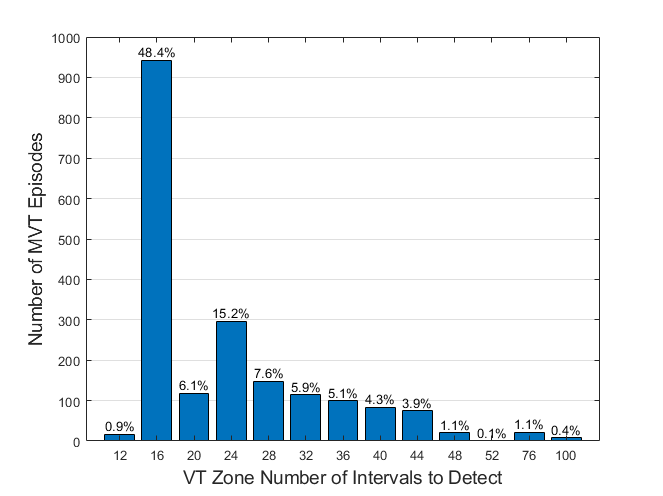


Figure S2: Histogram of the programmed VT Number of Intervals to Detect for MVT episodes. The percentage on the top of each bar is the proportion of the total number of MVT episodes at that initial detection setting in the VT zone (including FVT via VT). The nominal value is 16.

FIGURE S3:

Figure S3: Histogram of the number of configurations programmed with a given VF Detection Interval in devices with MVT episodes. The nominal value of 320ms is the second most frequently used value.

FIGURE S4:

Figure S4: Histogram of the programmed VF Number of Intervals to Detect for MVT episodes. The percentage on the top of each bar is the proportion of the total number of MVT episodes at that initial detection setting in the VF zone (including FVT via VF). The nominal value is 30/40.

FIGURE S5:


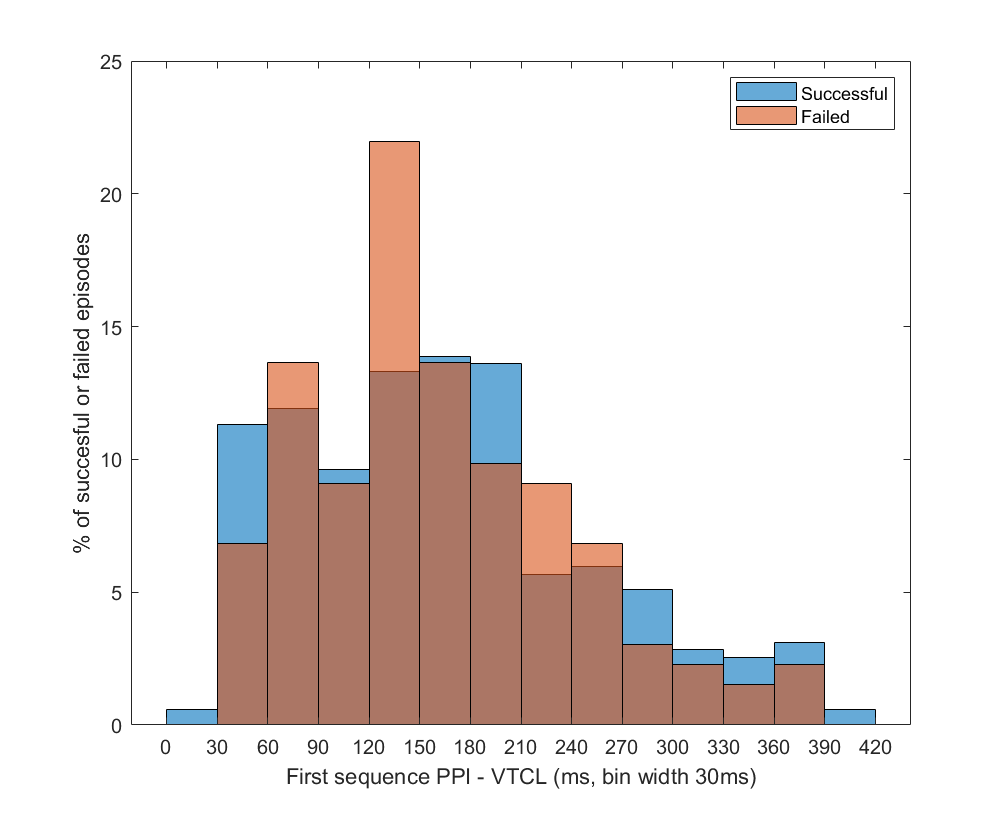


Figure S5: Distribution of the difference between the post-pacing interval (PPI) and the ventricular tachycardia cycle length (VTCL) following failed first IATP sequences. Because there were over four times more successful episodes than failed episodes the y-axis is normalized as the percentage within each group of successful and failed episodes for each cycle length bin.

**TABLES**

**Table S1: Mapping of indication for device to key terms.**

| **Assigned indication** | **Term in patient record** |
| --- | --- |
| Secondary prevention | "SECONDARY PREVENTION - PT SHOCKED PREV" |
|  | "PRIOR SUDDEN CARDIAC ARREST/VF" |
|  | "HISTORY OF SPONTANEOUS SUSTAINED VT" |
|  | "SYNCOPE WITH INDUCIBLE VT/VF" |
| Primary prevention | "PRIMARY PREVENTION - PT NEVER SHOCKED" |
|  | "DILATED CARDIOMYOPATHY" |
|  | "CARDIOMYOPATHY" |
|  | "HOCM (HYPERTROPHIC OBSTR CARDIOMYOPATHY)" |
|  | "LOW EF, DYSSYNCHRONY (WIDE QRS)" |
|  | "LOW EF, NO HISTORY VT/VF (SCD-HEFT)" |
|  | "NYHA CLASS III" |
| Other indication | "OTHER" |
|  | "SYNCOPE" |

**Table S2: Programming of FVT Sub-zone Boundaries**

| **FVT via VF - minimum interval for FVT classification** | **Number of patients with via VF interval** | **FVT via VT – maximum interval for VT classification** | **Number of patients with via VT interval** |
| --- | --- | --- | --- |
| 200 ms | 1 | 300 ms | 1 |
| 240 ms (nominal via VF) | 15 (44%) | 320 ms | 1 |
| 250 ms | 2 | 330 ms | 2 |
| 260 ms | 4 | 350 ms | 1 |
| 270 ms | 5 | 410 ms | 1 |
| 280 ms | 4 | 430 ms | 1 |
| 290 ms | 3 |  |  |

**Table S3: Programming of Minimum S2/S3 for Patients with Treated MVT in a Zone**

| **Minimum S2/S3 (ms)** | **VF Zone (71 configurations)** | **FVT zone (47 configurations)** | **VT Zone (268 configurations)** |
| --- | --- | --- | --- |
| **150** | 4% | 2% | 5% |
| **160** | 3% | 96% (nominal) | 92% (nominal) |
| **170** | 93% (nominal) | 2% | 0.4% |
| **180** | 0% | 0% | 3% |
